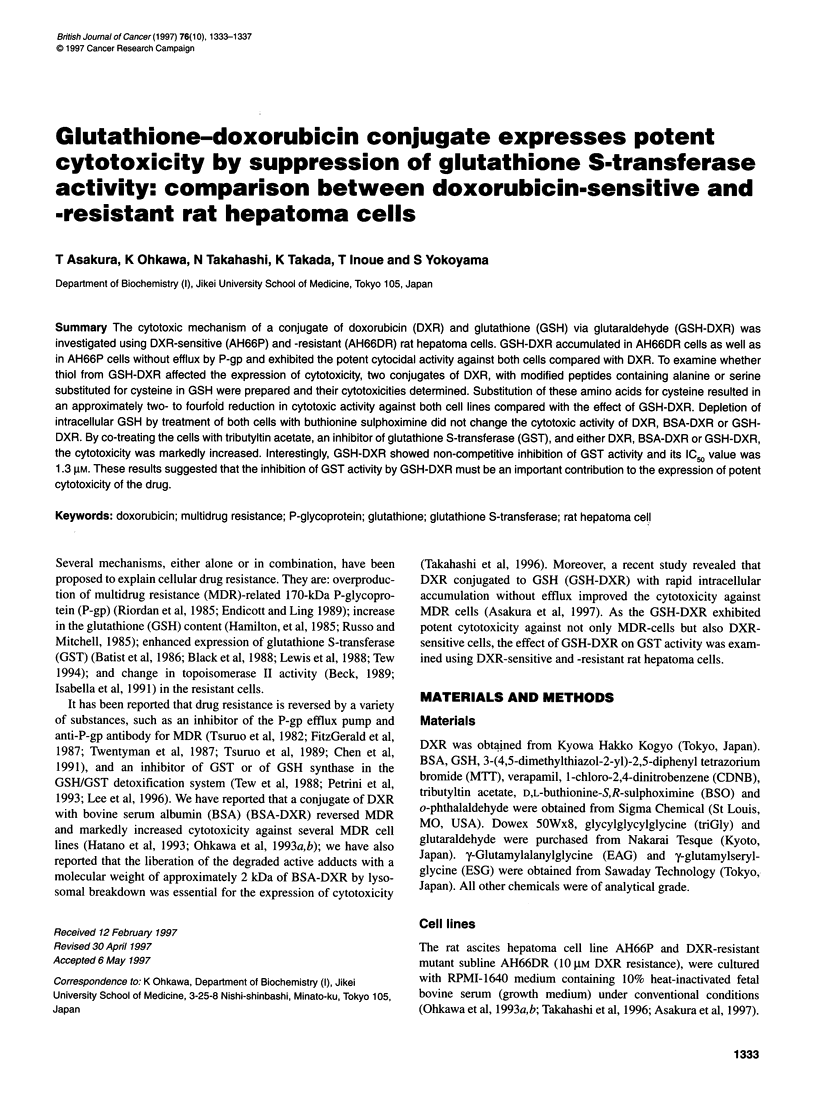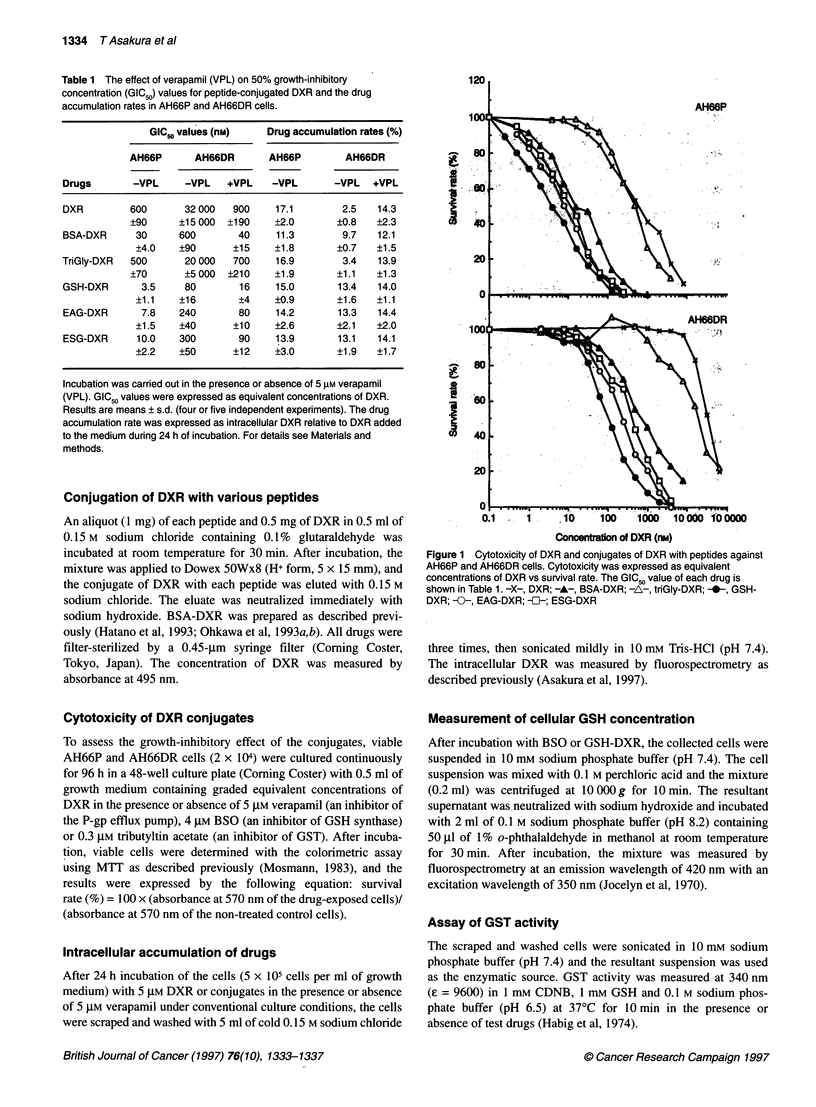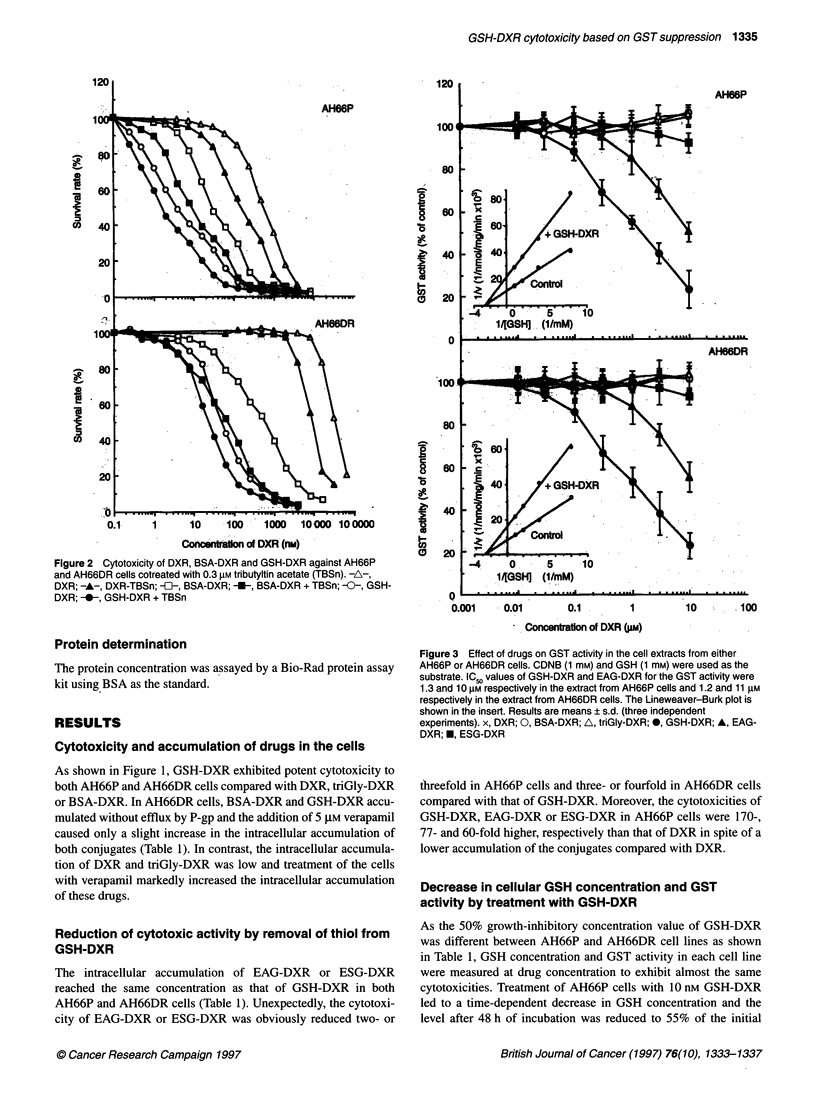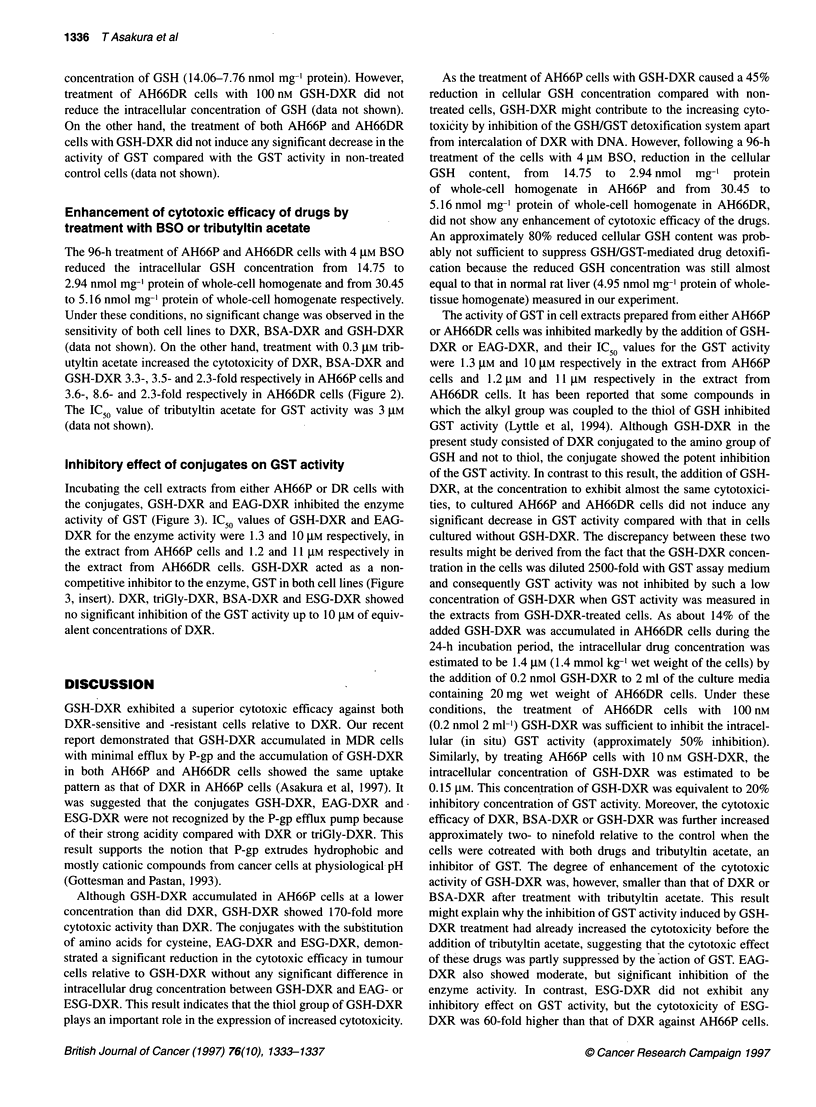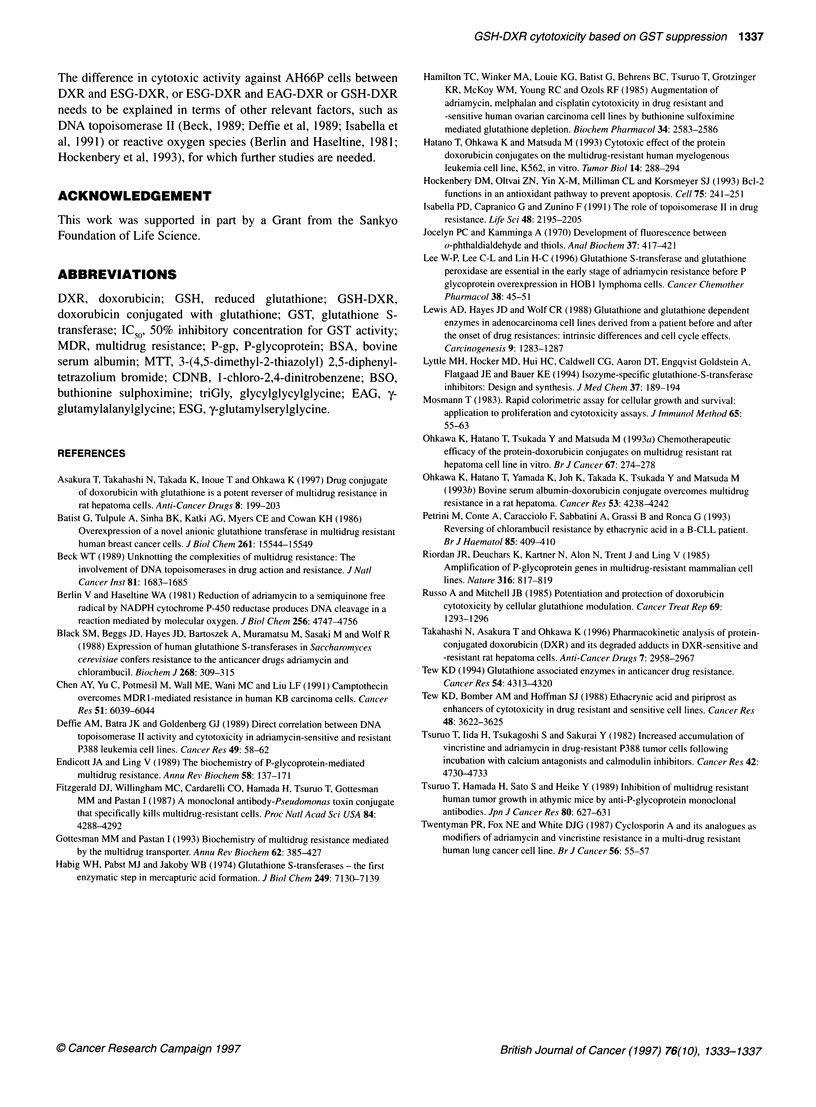# Glutathione-doxorubicin conjugate expresses potent cytotoxicity by suppression of glutathione S-transferase activity: comparison between doxorubicin-sensitive and -resistant rat hepatoma cells.

**DOI:** 10.1038/bjc.1997.557

**Published:** 1997

**Authors:** T. Asakura, K. Ohkawa, N. Takahashi, K. Takada, T. Inoue, S. Yokoyama

**Affiliations:** Department of Biochemistry (I), Jikei University School of Medicine, Tokyo, Japan.

## Abstract

The cytotoxic mechanism of a conjugate of doxorubicin (DXR) and glutathione (GSH) via glutaraldehyde (GSH-DXR) was investigated using DXR-sensitive (AH66P) and -resistant (AH66DR) rat hepatoma cells. GSH-DXR accumulated in AH66DR cells as well as in AH66P cells without efflux by P-gp and exhibited the potent cytocidal activity against both cells compared with DXR. To examine whether thiol from GSH-DXR affected the expression of cytotoxicity, two conjugates of DXR, with modified peptides containing alanine or serine substituted for cysteine in GSH were prepared and their cytotoxicities determined. Substitution of these amino acids for cysteine resulted in an approximately two- to fourfold reduction in cytotoxic activity against both cell lines compared with the effect of GSH-DXR. Depletion of intracellular GSH by treatment of both cells with buthionine sulphoximine did not change the cytotoxic activity of DXR, BSA-DXR or GSH-DXR. By co-treating the cells with tributyltin acetate, an inhibitor of glutathione S-transferase (GST), and either DXR, BSA-DXR or GSH-DXR, the cytotoxicity was markedly increased. Interestingly, GSH-DXR showed non-competitive inhibition of GST activity and its IC50 value was 1.3 microM. These results suggested that the inhibition of GST activity by GSH-DXR must be an important contribution to the expression of potent cytotoxicity of the drug.


British Journal of Cancer (1997) 76(10), 1333–1337
© 1997 Cancer Research Campaign

### Glutathione–doxorubicin conjugate expresses potent cytotoxicity by suppression of glutathione S-transferase activity: comparison between doxorubicin-sensitive and -resistant rat hepatoma cells

**T Asakura, K Ohkawa, N Takahashi, K Takada, T Inoue and S Yokoyama**

Department of Biochemistry (I), Jikei University School of Medicine, Tokyo 105, Japan

**Summary** The cytotoxic mechanism of a conjugate of doxorubicin (DXR) and glutathione (GSH) via glutaraldehyde (GSH-DXR) was investigated using DXR-sensitive (AH66P) and -resistant (AH66DR) rat hepatoma cells. GSH-DXR accumulated in AH66DR cells as well as in AH66P cells without efflux by P-gp and exhibited the potent cytocidal activity against both cells compared with DXR. To examine whether thiol from GSH-DXR affected the expression of cytotoxicity, two conjugates of DXR, with modified peptides containing alanine or serine substituted for cysteine in GSH were prepared and their cytotoxicities determined. Substitution of these amino acids for cysteine resulted in an approximately two- to fourfold reduction in cytotoxic activity against both cell lines compared with the effect of GSH-DXR. Depletion of intracellular GSH by treatment of both cells with buthionine sulphoximine did not change the cytotoxic activity of DXR, BSA-DXR or GSH-DXR. By co-treating the cells with tributyltin acetate, an inhibitor of glutathione S-transferase (GST), and either DXR, BSA-DXR or GSH-DXR, the cytotoxicity was markedly increased. Interestingly, GSH-DXR showed non-competitive inhibition of GST activity and its $IC_{50}$ value was 1.3 μM. These results suggested that the inhibition of GST activity by GSH-DXR must be an important contribution to the expression of potent cytotoxicity of the drug.

**Keywords:** doxorubicin; multidrug resistance; P-glycoprotein; glutathione; glutathione S-transferase; rat hepatoma cell

Several mechanisms, either alone or in combination, have been proposed to explain cellular drug resistance. They are: overproduction of multidrug resistance (MDR)-related 170-kDa P-glycoprotein (P-gp) (Riordan et al, 1985; Endicott and Ling 1989); increase in the glutathione (GSH) content (Hamilton, et al, 1985; Russo and Mitchell, 1985); enhanced expression of glutathione S-transferase (GST) (Batist et al, 1986; Black et al, 1988; Lewis et al, 1988; Tew 1994); and change in topoisomerase II activity (Beck, 1989; Isabella et al, 1991) in the resistant cells.

It has been reported that drug resistance is reversed by a variety of substances, such as an inhibitor of the P-gp efflux pump and anti-P-gp antibody for MDR (Tsuruo et al, 1982; FitzGerald et al, 1987; Twentyman et al, 1987; Tsuruo et al, 1989; Chen et al, 1991), and an inhibitor of GST or of GSH synthase in the GSH/GST detoxification system (Tew et al, 1988; Petrini et al, 1993; Lee et al, 1996). We have reported that a conjugate of DXR with bovine serum albumin (BSA) (BSA-DXR) reversed MDR and markedly increased cytotoxicity against several MDR cell lines (Hatano et al, 1993; Ohkawa et al, 1993*a,b*); we have also reported that the liberation of the degraded active adducts with a molecular weight of approximately 2 kDa of BSA-DXR by lysosomal breakdown was essential for the expression of cytotoxicity (Takahashi et al, 1996). Moreover, a recent study revealed that DXR conjugated to GSH (GSH-DXR) with rapid intracellular accumulation without efflux improved the cytotoxicity against MDR cells (Asakura et al, 1997). As the GSH-DXR exhibited potent cytotoxicity against not only MDR-cells but also DXR-sensitive cells, the effect of GSH-DXR on GST activity was examined using DXR-sensitive and -resistant rat hepatoma cells.

*Received 12 February 1997*
*Revised 30 April 1997*
*Accepted 6 May 1997*

*Correspondence to:* K Ohkawa, Department of Biochemistry (I), Jikei University School of Medicine, 3-25-8 Nishi-shinbashi, Minato-ku, Tokyo 105, Japan

### MATERIALS AND METHODS

### Materials

DXR was obtained from Kyowa Hakko Kogyo (Tokyo, Japan). BSA, GSH, 3-(4,5-dimethylthiazol-2-yl)-2,5-diphenyl tetrazorium bromide (MTT), verapamil, 1-chloro-2,4-dinitrobenzene (CDNB), tributyltin acetate, D,L-buthionine-*S,R*-sulphoximine (BSO) and *o*-phthalaldehyde were obtained from Sigma Chemical (St Louis, MO, USA). Dowex 50Wx8, glycylglycylglycine (triGly) and glutaraldehyde were purchased from Nakarai Tesque (Kyoto, Japan). γ-Glutamylalanylglycine (EAG) and γ-glutamylserylglycine (ESG) were obtained from Sawaday Technology (Tokyo, Japan). All other chemicals were of analytical grade.

### Cell lines

The rat ascites hepatoma cell line AH66P and DXR-resistant mutant subline AH66DR (10 μM DXR resistance), were cultured with RPMI-1640 medium containing 10% heat-inactivated fetal bovine serum (growth medium) under conventional conditions (Ohkawa et al, 1993*a,b*; Takahashi et al, 1996; Asakura et al, 1997).

**Table 1** The effect of verapamil (VPL) on 50% growth-inhibitory concentration ($GIC_{50}$) values for peptide-conjugated DXR and the drug accumulation rates in AH66P and AH66DR cells.

| | $GIC_{50}$ values (nM) | | | Drug accumulation rates (%) | | |
|---|---|---|---|---|---|---|
| | AH66P | AH66DR | | AH66P | AH66DR | |
| Drugs | –VPL | –VPL | +VPL | –VPL | –VPL | +VPL |
| DXR | 600 | 32 000 | 900 | 17.1 | 2.5 | 14.3 |
| | ±90 | ±15 000 | ±190 | ±2.0 | ±0.8 | ±2.3 |
| BSA-DXR | 30 | 600 | 40 | 11.3 | 9.7 | 12.1 |
| | ±4.0 | ±90 | ±15 | ±1.8 | ±0.7 | ±1.5 |
| TriGly-DXR | 500 | 20 000 | 700 | 16.9 | 3.4 | 13.9 |
| | ±70 | ±5 000 | ±210 | ±1.9 | ±1.1 | ±1.3 |
| GSH-DXR | 3.5 | 80 | 16 | 15.0 | 13.4 | 14.0 |
| | ±1.1 | ±16 | ±4 | ±0.9 | ±1.6 | ±1.1 |
| EAG-DXR | 7.8 | 240 | 80 | 14.2 | 13.3 | 14.4 |
| | ±1.5 | ±40 | ±10 | ±2.6 | ±2.1 | ±2.0 |
| ESG-DXR | 10.0 | 300 | 90 | 13.9 | 13.1 | 14.1 |
| | ±2.2 | ±50 | ±12 | ±3.0 | ±1.9 | ±1.7 |

Incubation was carried out in the presence or absence of 5 μM verapamil (VPL). $GIC_{50}$ values were expressed as equivalent concentrations of DXR. Results are means ± s.d. (four or five independent experiments). The drug accumulation rate was expressed as intracellular DXR relative to DXR added to the medium during 24 h of incubation. For details see Materials and methods.

**Figure 1** Cytotoxicity of DXR and conjugates of DXR with peptides against AH66P and AH66DR cells. Cytotoxicity was expressed as equivalent concentrations of DXR vs survival rate. The $GIC_{50}$ value of each drug is shown in Table 1. –X–, DXR; –▲–, BSA-DXR; –△–, triGly-DXR; –●–, GSH-DXR; –○–, EAG-DXR; –□–; ESG-DXR

### Conjugation of DXR with various peptides

An aliquot (1 mg) of each peptide and 0.5 mg of DXR in 0.5 ml of 0.15 M sodium chloride containing 0.1% glutaraldehyde was incubated at room temperature for 30 min. After incubation, the mixture was applied to Dowex 50Wx8 ($H^+$ form, 5 × 15 mm), and the conjugate of DXR with each peptide was eluted with 0.15 M sodium chloride. The eluate was neutralized immediately with sodium hydroxide. BSA-DXR was prepared as described previously (Hatano et al, 1993; Ohkawa et al, 1993*a,b*). All drugs were filter-sterilized by a 0.45-μm syringe filter (Corning Coster, Tokyo, Japan). The concentration of DXR was measured by absorbance at 495 nm.

### Cytotoxicity of DXR conjugates

To assess the growth-inhibitory effect of the conjugates, viable AH66P and AH66DR cells (2 × $10^4$) were cultured continuously for 96 h in a 48-well culture plate (Corning Coster) with 0.5 ml of growth medium containing graded equivalent concentrations of DXR in the presence or absence of 5 μM verapamil (an inhibitor of the P-gp efflux pump), 4 μM BSO (an inhibitor of GSH synthase) or 0.3 μM tributyltin acetate (an inhibitor of GST). After incubation, viable cells were determined with the colorimetric assay using MTT as described previously (Mosmann, 1983), and the results were expressed by the following equation: survival rate (%) = 100 × (absorbance at 570 nm of the drug-exposed cells)/(absorbance at 570 nm of the non-treated control cells).

### Intracellular accumulation of drugs

After 24 h incubation of the cells (5 × $10^5$ cells per ml of growth medium) with 5 μM DXR or conjugates in the presence or absence of 5 μM verapamil under conventional culture conditions, the cells were scraped and washed with 5 ml of cold 0.15 M sodium chloride three times, then sonicated mildly in 10 mM Tris-HCl (pH 7.4). The intracellular DXR was measured by fluorospectrometry as described previously (Asakura et al, 1997).

### Measurement of cellular GSH concentration

After incubation with BSO or GSH-DXR, the collected cells were suspended in 10 mM sodium phosphate buffer (pH 7.4). The cell suspension was mixed with 0.1 M perchloric acid and the mixture (0.2 ml) was centrifuged at 10 000 *g* for 10 min. The resultant supernatant was neutralized with sodium hydroxide and incubated with 2 ml of 0.1 M sodium phosphate buffer (pH 8.2) containing 50 μl of 1% *o*-phthalaldehyde in methanol at room temperature for 30 min. After incubation, the mixture was measured by fluorospectrometry at an emission wavelength of 420 nm with an excitation wavelength of 350 nm (Jocelyn et al, 1970).

### Assay of GST activity

The scraped and washed cells were sonicated in 10 mM sodium phosphate buffer (pH 7.4) and the resultant suspension was used as the enzymatic source. GST activity was measured at 340 nm (ε = 9600) in 1 mM CDNB, 1 mM GSH and 0.1 M sodium phosphate buffer (pH 6.5) at 37°C for 10 min in the presence or absence of test drugs (Habig et al, 1974).

 © Cancer Research Campaign 1997

**Figure 2** Cytotoxicity of DXR, BSA-DXR and GSH-DXR against AH66P and AH66DR cells cotreated with 0.3 μM tributyltin acetate (TBSn). -△-, DXR; -▲-, DXR-TBSn; -□-, BSA-DXR; -■-, BSA-DXR + TBSn; -○-, GSH-DXR; -●-, GSH-DXR + TBSn

**Figure 3** Effect of drugs on GST activity in the cell extracts from either AH66P or AH66DR cells. CDNB (1 mM) and GSH (1 mM) were used as the substrate. $IC_{50}$ values of GSH-DXR and EAG-DXR for the GST activity were 1.3 and 10 μM respectively in the extract from AH66P cells and 1.2 and 11 μM respectively in the extract from AH66DR cells. The Lineweaver–Burk plot is shown in the insert. Results are means ± s.d. (three independent experiments). ×, DXR; ○, BSA-DXR; △, triGly-DXR; ●, GSH-DXR; ▲, EAG-DXR; ■, ESG-DXR

### Protein determination

The protein concentration was assayed by a Bio-Rad protein assay kit using BSA as the standard.

### RESULTS

### Cytotoxicity and accumulation of drugs in the cells

As shown in Figure 1, GSH-DXR exhibited potent cytotoxicity to both AH66P and AH66DR cells compared with DXR, triGly-DXR or BSA-DXR. In AH66DR cells, BSA-DXR and GSH-DXR accumulated without efflux by P-gp and the addition of 5 μM verapamil caused only a slight increase in the intracellular accumulation of both conjugates (Table 1). In contrast, the intracellular accumulation of DXR and triGly-DXR was low and treatment of the cells with verapamil markedly increased the intracellular accumulation of these drugs.

### Reduction of cytotoxic activity by removal of thiol from GSH-DXR

The intracellular accumulation of EAG-DXR or ESG-DXR reached the same concentration as that of GSH-DXR in both AH66P and AH66DR cells (Table 1). Unexpectedly, the cytotoxicity of EAG-DXR or ESG-DXR was obviously reduced two- or threefold in AH66P cells and three- or fourfold in AH66DR cells compared with that of GSH-DXR. Moreover, the cytotoxicities of GSH-DXR, EAG-DXR or ESG-DXR in AH66P cells were 170-, 77- and 60-fold higher, respectively than that of DXR in spite of a lower accumulation of the conjugates compared with DXR.

### Decrease in cellular GSH concentration and GST activity by treatment with GSH-DXR

As the 50% growth-inhibitory concentration value of GSH-DXR was different between AH66P and AH66DR cell lines as shown in Table 1, GSH concentration and GST activity in each cell line were measured at drug concentration to exhibit almost the same cytotoxicities. Treatment of AH66P cells with 10 nM GSH-DXR led to a time-dependent decrease in GSH concentration and the level after 48 h of incubation was reduced to 55% of the initial

© Cancer Research Campaign 1997

concentration of GSH (14.06–7.76 nmol $mg^{-1}$ protein). However, treatment of AH66DR cells with 100 nM GSH-DXR did not reduce the intracellular concentration of GSH (data not shown). On the other hand, the treatment of both AH66P and AH66DR cells with GSH-DXR did not induce any significant decrease in the activity of GST compared with the GST activity in non-treated control cells (data not shown).

### Enhancement of cytotoxic efficacy of drugs by treatment with BSO or tributyltin acetate

The 96-h treatment of AH66P and AH66DR cells with 4 μM BSO reduced the intracellular GSH concentration from 14.75 to 2.94 nmol $mg^{-1}$ protein of whole-cell homogenate and from 30.45 to 5.16 nmol $mg^{-1}$ protein of whole-cell homogenate respectively. Under these conditions, no significant change was observed in the sensitivity of both cell lines to DXR, BSA-DXR and GSH-DXR (data not shown). On the other hand, treatment with 0.3 μM tributyltin acetate increased the cytotoxicity of DXR, BSA-DXR and GSH-DXR 3.3-, 3.5- and 2.3-fold respectively in AH66P cells and 3.6-, 8.6- and 2.3-fold respectively in AH66DR cells (Figure 2). The $IC_{50}$ value of tributyltin acetate for GST activity was 3 μM (data not shown).

### Inhibitory effect of conjugates on GST activity

Incubating the cell extracts from either AH66P or DR cells with the conjugates, GSH-DXR and EAG-DXR inhibited the enzyme activity of GST (Figure 3). $IC_{50}$ values of GSH-DXR and EAG-DXR for the enzyme activity were 1.3 and 10 μM respectively, in the extract from AH66P cells and 1.2 and 11 μM respectively in the extract from AH66DR cells. GSH-DXR acted as a non-competitive inhibitor to the enzyme, GST in both cell lines (Figure 3, insert). DXR, triGly-DXR, BSA-DXR and ESG-DXR showed no significant inhibition of the GST activity up to 10 μM of equivalent concentrations of DXR.

### DISCUSSION

GSH-DXR exhibited a superior cytotoxic efficacy against both DXR-sensitive and -resistant cells relative to DXR. Our recent report demonstrated that GSH-DXR accumulated in MDR cells with minimal efflux by P-gp and the accumulation of GSH-DXR in both AH66P and AH66DR cells showed the same uptake pattern as that of DXR in AH66P cells (Asakura et al, 1997). It was suggested that the conjugates GSH-DXR, EAG-DXR and ESG-DXR were not recognized by the P-gp efflux pump because of their strong acidity compared with DXR or triGly-DXR. This result supports the notion that P-gp extrudes hydrophobic and mostly cationic compounds from cancer cells at physiological pH (Gottesman and Pastan, 1993).

Although GSH-DXR accumulated in AH66P cells at a lower concentration than did DXR, GSH-DXR showed 170-fold more cytotoxic activity than DXR. The conjugates with the substitution of amino acids for cysteine, EAG-DXR and ESG-DXR, demonstrated a significant reduction in the cytotoxic efficacy in tumour cells relative to GSH-DXR without any significant difference in intracellular drug concentration between GSH-DXR and EAG- or ESG-DXR. This result indicates that the thiol group of GSH-DXR plays an important role in the expression of increased cytotoxicity.

As the treatment of AH66P cells with GSH-DXR caused a 45% reduction in cellular GSH concentration compared with non-treated cells, GSH-DXR might contribute to the increasing cytotoxicity by inhibition of the GSH/GST detoxification system apart from intercalation of DXR with DNA. However, following a 96-h treatment of the cells with 4 μM BSO, reduction in the cellular GSH content, from 14.75 to 2.94 nmol $mg^{-1}$ protein of whole-cell homogenate in AH66P and from 30.45 to 5.16 nmol $mg^{-1}$ protein of whole-cell homogenate in AH66DR, did not show any enhancement of cytotoxic efficacy of the drugs. An approximately 80% reduced cellular GSH content was probably not sufficient to suppress GSH/GST-mediated drug detoxification because the reduced GSH concentration was still almost equal to that in normal rat liver (4.95 nmol $mg^{-1}$ protein of whole-tissue homogenate) measured in our experiment.

The activity of GST in cell extracts prepared from either AH66P or AH66DR cells was inhibited markedly by the addition of GSH-DXR or EAG-DXR, and their $IC_{50}$ values for the GST activity were 1.3 μM and 10 μM respectively in the extract from AH66P cells and 1.2 μM and 11 μM respectively in the extract from AH66DR cells. It has been reported that some compounds in which the alkyl group was coupled to the thiol of GSH inhibited GST activity (Lyttle et al, 1994). Although GSH-DXR in the present study consisted of DXR conjugated to the amino group of GSH and not to thiol, the conjugate showed the potent inhibition of the GST activity. In contrast to this result, the addition of GSH-DXR, at the concentration to exhibit almost the same cytotoxicities, to cultured AH66P and AH66DR cells did not induce any significant decrease in GST activity compared with that in cells cultured without GSH-DXR. The discrepancy between these two results might be derived from the fact that the GSH-DXR concentration in the cells was diluted 2500-fold with GST assay medium and consequently GST activity was not inhibited by such a low concentration of GSH-DXR when GST activity was measured in the extracts from GSH-DXR-treated cells. As about 14% of the added GSH-DXR was accumulated in AH66DR cells during the 24-h incubation period, the intracellular drug concentration was estimated to be 1.4 μM (1.4 mmol $kg^{-1}$ wet weight of the cells) by the addition of 0.2 nmol GSH-DXR to 2 ml of the culture media containing 20 mg wet weight of AH66DR cells. Under these conditions, the treatment of AH66DR cells with 100 nM (0.2 nmol 2 $ml^{-1}$) GSH-DXR was sufficient to inhibit the intracellular (in situ) GST activity (approximately 50% inhibition). Similarly, by treating AH66P cells with 10 nM GSH-DXR, the intracellular concentration of GSH-DXR was estimated to be 0.15 μM. This concentration of GSH-DXR was equivalent to 20% inhibitory concentration of GST activity. Moreover, the cytotoxic efficacy of DXR, BSA-DXR or GSH-DXR was further increased approximately two- to ninefold relative to the control when the cells were cotreated with both drugs and tributyltin acetate, an inhibitor of GST. The degree of enhancement of the cytotoxic activity of GSH-DXR was, however, smaller than that of DXR or BSA-DXR after treatment with tributyltin acetate. This result might explain why the inhibition of GST activity induced by GSH-DXR treatment had already increased the cytotoxicity before the addition of tributyltin acetate, suggesting that the cytotoxic effect of these drugs was partly suppressed by the action of GST. EAG-DXR also showed moderate, but significant inhibition of the enzyme activity. In contrast, ESG-DXR did not exhibit any inhibitory effect on GST activity, but the cytotoxicity of ESG-DXR was 60-fold higher than that of DXR against AH66P cells.

© Cancer Research Campaign 1997

The difference in cytotoxic activity against AH66P cells between DXR and ESG-DXR, or ESG-DXR and EAG-DXR or GSH-DXR needs to be explained in terms of other relevant factors, such as DNA topoisomerase II (Beck, 1989; Deffie et al, 1989; Isabella et al, 1991) or reactive oxygen species (Berlin and Haseltine, 1981; Hockenbery et al, 1993), for which further studies are needed.

### ACKNOWLEDGEMENT

This work was supported in part by a Grant from the Sankyo Foundation of Life Science.

### ABBREVIATIONS

DXR, doxorubicin; GSH, reduced glutathione; GSH-DXR, doxorubicin conjugated with glutathione; GST, glutathione S-transferase; $IC_{50}$, 50% inhibitory concentration for GST activity; MDR, multidrug resistance; P-gp, P-glycoprotein; BSA, bovine serum albumin; MTT, 3-(4,5-dimethyl-2-thiazolyl) 2,5-diphenyl-tetrazolium bromide; CDNB, 1-chloro-2,4-dinitrobenzene; BSO, buthionine sulphoximine; triGly, glycylglycylglycine; EAG, γ-glutamylalanylglycine; ESG, γ-glutamylserylglycine.

### REFERENCES

Asakura T, Takahashi N, Takada K, Inoue T and Ohkawa K (1997) Drug conjugate of doxorubicin with glutathione is a potent reverser of multidrug resistance in rat hepatoma cells. *Anti-Cancer Drugs* **8**: 199–203

Batist G, Tulpule A, Sinha BK, Katki AG, Myers CE and Cowan KH (1986) Overexpression of a novel anionic glutathione transferase in multidrug resistant human breast cancer cells. *J Biol Chem* **261**: 15544–15549

Beck WT (1989) Unknotting the complexities of multidrug resistance: The involvement of DNA topoisomerases in drug action and resistance. *J Natl Cancer Inst* **81**: 1683–1685

Berlin V and Haseltine WA (1981) Reduction of adriamycin to a semiquinone free radical by NADPH cytochrome P-450 reductase produces DNA cleavage in a reaction mediated by molecular oxygen. *J Biol Chem* **256**: 4747–4756

Black SM, Beggs JD, Hayes JD, Bartoszek A, Muramatsu M, Sasaki M and Wolf R (1988) Expression of human glutathione S-transferases in *Saccharomyces cerevisiae* confers resistance to the anticancer drugs adriamycin and chlorambucil. *Biochem J* **268**: 309–315

Chen AY, Yu C, Potmesil M, Wall ME, Wani MC and Liu LF (1991) Camptothecin overcomes MDR1-mediated resistance in human KB carcinoma cells. *Cancer Res* **51**: 6039–6044

Deffie AM, Batra JK and Goldenberg GJ (1989) Direct correlation between DNA topoisomerase II activity and cytotoxicity in adriamycin-sensitive and resistant P388 leukemia cell lines. *Cancer Res* **49**: 58–62

Endicott JA and Ling V (1989) The biochemistry of P-glycoprotein-mediated multidrug resistance. *Annu Rev Biochem* **58**: 137–171

Fitzgerald DJ, Willingham MC, Cardarelli CO, Hamada H, Tsuruo T, Gottesman MM and Pastan I (1987) A monoclonal antibody-*Pseudomonas* toxin conjugate that specifically kills multidrug-resistant cells. *Proc Natl Acad Sci USA* **84**: 4288–4292

Gottesman MM and Pastan I (1993) Biochemistry of multidrug resistance mediated by the multidrug transporter. *Annu Rev Biochem* **62**: 385–427

Habig WH, Pabst MJ and Jakoby WB (1974) Glutathione S-transferases – the first enzymatic step in mercapturic acid formation. *J Biol Chem* **249**: 7130–7139

Hamilton TC, Winker MA, Louie KG, Batist G, Behrens BC, Tsuruo T, Grotzinger KR, McKoy WM, Young RC and Ozols RF (1985) Augmentation of adriamycin, melphalan and cisplatin cytotoxicity in drug resistant and -sensitive human ovarian carcinoma cell lines by buthionine sulfoximine mediated glutathione depletion. *Biochem Pharmacol* **34**: 2583–2586

Hatano T, Ohkawa K and Matsuda M (1993) Cytotoxic effect of the protein doxorubicin conjugates on the multidrug-resistant human myelogenous leukemia cell line, K562, in vitro. *Tumor Biol* **14**: 288–294

Hockenbery DM, Oltvai ZN, Yin X-M, Milliman CL and Korsmeyer SJ (1993) Bcl-2 functions in an antioxidant pathway to prevent apoptosis. *Cell* **75**: 241–251

Isabella PD, Capranico G and Zunino F (1991) The role of topoisomerase II in drug resistance. *Life Sci* **48**: 2195–2205

Jocelyn PC and Kamminga A (1970) Development of fluorescence between *o*-phthaldialdehyde and thiols. *Anal Biochem* **37**: 417–421

Lee W-P, Lee C-L and Lin H-C (1996) Glutathione S-transferase and glutathione peroxidase are essential in the early stage of adriamycin resistance before P glycoprotein overexpression in HOB1 lymphoma cells. *Cancer Chemother Pharmacol* **38**: 45–51

Lewis AD, Hayes JD and Wolf CR (1988) Glutathione and glutathione dependent enzymes in adenocarcinoma cell lines derived from a patient before and after the onset of drug resistances: intrinsic differences and cell cycle effects. *Carcinogenesis* **9**: 1283–1287

Lyttle MH, Hocker MD, Hui HC, Caldwell CG, Aaron DT, Engqvist Goldstein A, Flatgaad JE and Bauer KE (1994) Isozyme-specific glutathione-S-transferase inhibitors: Design and synthesis. *J Med Chem* **37**: 189–194

Mosmann T (1983). Rapid colorimetric assay for cellular growth and survival: application to proliferation and cytotoxicity assays. *J Immunol Method* **65**: 55–63

Ohkawa K, Hatano T, Tsukada Y and Matsuda M (1993*a*) Chemotherapeutic efficacy of the protein-doxorubicin conjugates on multidrug resistant rat hepatoma cell line in vitro. *Br J Cancer* **67**: 274–278

Ohkawa K, Hatano T, Yamada K, Joh K, Takada K, Tsukada Y and Matsuda M (1993*b*) Bovine serum albumin-doxorubicin conjugate overcomes multidrug resistance in a rat hepatoma. *Cancer Res* **53**: 4238–4242

Petrini M, Conte A, Caracciolo F, Sabbatini A, Grassi B and Ronca G (1993) Reversing of chlorambucil resistance by ethacrynic acid in a B-CLL patient. *Br J Haematol* **85**: 409–410

Riordan JR, Deuchars K, Kartner N, Alon N, Trent J and Ling V (1985) Amplification of P-glycoprotein genes in multidrug-resistant mammalian cell lines. *Nature* **316**: 817–819

Russo A and Mitchell JB (1985) Potentiation and protection of doxorubicin cytotoxicity by cellular glutathione modulation. *Cancer Treat Rep* **69**: 1293–1296

Takahashi N, Asakura T and Ohkawa K (1996) Pharmacokinetic analysis of protein-conjugated doxorubicin (DXR) and its degraded adducts in DXR-sensitive and -resistant rat hepatoma cells. *Anti-Cancer Drugs* **7**: 2958–2967

Tew KD (1994) Glutathione associated enzymes in anticancer drug resistance. *Cancer Res* **54**: 4313–4320

Tew KD, Bomber AM and Hoffman SJ (1988) Ethacrynic acid and piriprost as enhancers of cytotoxicity in drug resistant and sensitive cell lines. *Cancer Res* **48**: 3622–3625

Tsuruo T, Iida H, Tsukagoshi S and Sakurai Y (1982) Increased accumulation of vincristine and adriamycin in drug-resistant P388 tumor cells following incubation with calcium antagonists and calmodulin inhibitors. *Cancer Res* **42**: 4730–4733

Tsuruo T, Hamada H, Sato S and Heike Y (1989) Inhibition of multidrug resistant human tumor growth in athymic mice by anti-P-glycoprotein monoclonal antibodies. *Jpn J Cancer Res* **80**: 627–631

Twentyman PR, Fox NE and White DJG (1987) Cyclosporin A and its analogues as modifiers of adriamycin and vincristine resistance in a multi-drug resistant human lung cancer cell line. *Br J Cancer* **56**: 55–57

© Cancer Research Campaign 1997